# TCDCA inhibits pyroptosis to alleviate sepsis-related acute hepatic injury via activating TGR5

**DOI:** 10.3389/fimmu.2025.1706041

**Published:** 2025-11-26

**Authors:** Yang Yuexiang, Rong Zhiwei, Baitian Li, Wang Qing, Liu Chunzheng, Wang Zetian, Liao Lijun

**Affiliations:** 1 Postgraduate Training Base of Jinzhou Medical University, Shanghai East Hospital, Shanghai, China; 2 Department of Pathology, Shanghai Public Health Clinical Center, Fudan University, Shanghai, China; 3 Department of Pain Management, Shanghai East Hospital, School of Medicine, Tongji University, Shanghai, China

**Keywords:** sepsis, acute hepatic injury, taurochenodeoxycholic acid, TGR5, hepatocyte pyroptosis

## Abstract

**Introduction:**

Sepsis-related acute liver injury (SALI) is a severe and life-threatening complication in septic patients, for which current therapeutic options are limited. This study aimed to investigate the potential protective role of taurochenodeoxycholic acid (TCDCA) against SALI and to elucidate the underlying mechanisms.

**Methods:**

A cecal ligation and puncture (CLP) mouse model was employed to induce SALI. The effects of TCDCA treatment were assessed by measuring serum liver injury markers (AST, ALT) and pro-inflammatory cytokines (IL-6, TNF-α, IL-1β). Liver histology, hepatocyte apoptosis, and the macrophage response were evaluated. Molecular docking was used to predict the interaction between TCDCA and the receptor TGR5, which was functionally validated using the TGR5 antagonist SBI-115. Transcriptomic analysis and Western blotting were performed to identify the key signaling pathways involved.

**Results:**

TCDCA treatment significantly reduced serum levels of AST and ALT, suppressed the production of IL-6, TNF-α, and IL-1β, and alleviated histological liver damage, including lobular disruption, inflammation, and hemorrhage. TCDCA also decreased hepatocyte apoptosis and modulated the liver macrophage response. Molecular docking confirmed a strong interaction between TCDCA and TGR5, and the protective effects of TCDCA were abolished by the TGR5 antagonist SBI-115. Transcriptomic analysis identified 430 differentially expressed genes after TCDCA treatment, with significant enrichment in pyroptosis-related pathways. Accordingly, Western blot analysis demonstrated that TCDCA inhibited the activation of the NLRP3 inflammasome and its downstream pyroptotic proteins, an effect that was also reversed by SBI-115.

**Discussion:**

Our findings demonstrate that TCDCA confers a protective effect against SALI by suppressing hepatocyte pyroptosis, and this action is mediated through the TGR5 receptor. These results highlight TCDCA as a promising therapeutic candidate for SALI. However, further research, including clinical trials, is necessary to address potential species-specific differences and to fully elucidate its comprehensive mechanisms of action.

## 1 Introduction

Sepsis-related acute Hepatic injury (SAILI) is a life-threatening complication in critically ill sepsis patients, characterized by sudden Hepatic function impairment (1). Sepsis-induced hypotension and disseminated intravascular coagulation cause hepatic microcirculatory disturbances and ischemia- reperfusion injury, as indicated by sinusoidal congestion and necrosis in histopathology, thus exacerbating Hepatic damage (2). Current treatments primarily involve source control of sepsis based on microbiological and clinical assessments, appropriate fluid resuscitation, correction of coagulation disorders (e.g., using fresh frozen plasma or vitamin K), and comprehensive supportive care (3). However, these interventions have limited impact on improving patient outcomes.

Bile acid metabolites have become a research focus in sepsis treatment (4, 5). Secondary bile acids, such as deoxycholic and lithocholic acids, activate the Farnesoid X receptor (FXR) to regulate acute - phase proteins and suppress pro - inflammatory cytokines, reducing systemic inflammation (6). Binding to G protein - coupled bile acid receptor 1 (TGR5) enhances macrophage anti - inflammatory activity and restores immune homeostasis. Modulating intestinal bile acid metabolite levels can prevent bacterial and endotoxin translocation, halting sepsis progression (7, 8). Emerging evidence has highlighted the pivotal protective role of TGR5 signaling activation in the pathophysiology of sepsis. Recent studies demonstrate that TGR5 activation not only drives the polarization of hepatic macrophages toward an anti-inflammatory phenotype but also directly suppresses the assembly and activation of the NLRP3 inflammasome, thereby mitigating hepatocyte pyroptosis and inflammatory damage (9). Furthermore, TGR5 plays a critical role in the gut-liver axis by maintaining intestinal barrier integrity and bile acid homeostasis, which in turn inhibits bacterial and endotoxin translocation and attenuates the progression of sepsis (10, 11). Our previous observations showed that taurochenodeoxycholic acid (TCDCA) levels were significantly reduced in a cecal ligation and puncture (CLP) sepsis model, and TCDCA treatment improved survival. However, its specific role in SALI remains unexplored.

Sepsis - triggered hepatocyte injury contributes to multi - organ failure and poor prognosis, with pyroptosis being a key factor in Hepatic injury (12). Pathogen - and damage - associated molecular patterns (PAMPs and DAMPs) activate pattern recognition receptors (PRRs), leading to NOD - like receptor protein 3 (NLRP3) inflammasome activation (13). Activated NLRP3 recruits caspase - 1 via ASC, which cleaves gasdermin D to form membrane - pore - forming fragments, releasing pro-inflammatory contents, and amplifying the inflammatory cascade. Caspase - 1 also promotes maturation of IL-1β and IL-18, worsening local and systemic inflammation (14).

This study aims to establish a mouse model of sepsis - induced Hepatic injury and use transcriptomic analysis to determine whether TCDCA can mitigate Hepatic injury by suppressing hepatocyte pyroptosis through TGR5, potentially uncovering new therapeutic strategies for sepsis.

## 2 Materials and methods

### 2.1 Mouse models

A total of 75 male C57BL/6 mice (8 weeks old, 20–25 g) were purchased from the Animal Center of Tongji University (Shanghai, China). The animals were housed in individually ventilated cages under controlled conditions (20–22°C, 12-h light/dark cycle) with free access to standard rodent chow and water. All experimental protocols were approved by the Institutional Animal Care and Use Committee of Tongji University. After a one-week acclimatization period, the mice were randomly divided into five groups (n = 15 per group): Sham, CLP (cecal ligation and puncture), CLP + TCDCA (200 mg/kg, Aladdin, Shanghai, China), CLP + TCDCA + SBI-115 (10 mg/kg), Guangzhou Bio-gene Technology Co., Ltd., China), and TCDCA alone. TCDCA was administered daily via oral gavage for seven days, while SBI-115 was delivered intraperitoneally once per day over the same period. On the seventh day, sepsis was induced via CLP surgery, and biological specimens were collected 24 hours later for subsequent analyses. Due to differential mortality across groups following CLP, the final number of survivors varied. To ensure unbiased statistical comparisons with equal sample sizes across all groups, a random subset of n=6 animals from each group was selected for all endpoint biochemical, molecular, and histological analyses.

### 2.2 Hepatic histomorphological analysis

Hepatic tissue specimens were immediately immersed in 10% neutral-buffered formalin solution in PBS for 24 hours to facilitate complete fixation. Following fixation, paraffin embedding was performed using a standardized protocol to preserve tissue integrity. The embedded tissues were sectioned into 4-μm slices with a microtome and subsequently stained with hematoxylin and eosin (H&E) to visualize cellular and histological features. Stained slides were examined, and images were captured under an Olympus CX30 light microscope (Olympus, Tokyo, Japan). Hepatic parenchymal changes, including necrosis, inflammatory infiltration, and architectural distortion, were evaluated by board-certified pathologists unaware of the experimental group assignments. This double-blind assessment ensured unbiased quantification of sepsis-induced Hepatic injury and the therapeutic efficacy of experimental interventions.

### 2.3 Quantitative PCR

Liver samples were harvested, snap-frozen immediately, and preserved at -80°C until use. Total RNA was isolated using Trizol reagent (cat. no. 15596026; Invitrogen, Carlsbad, CA, USA). Quantitative analysis was performed with the Universal SYBR FAST qPCR Master Mix (2×; KAPA Biosystems, Wilmington, MA, USA) following the thermal cycling protocol: initial denaturation at 95°C for 10 minutes, followed by 45 cycles of denaturation at 95°C for 10 seconds, annealing at 59°C for 60 seconds, and extension at 72°C for 15 seconds, with a final dissociation step at 95°C for 15 seconds. The qPCR assays utilized gene-specific oligonucleotide primer pairs (synthesized by Invitrogen, Shanghai, China) with sequences (5′ to 3′) as follows: TNF-α: forward 5′-GTTCTATGGCCCAGACCCTCAC-3′ and reverse 5′-GGCACCACTAGTTGGTTGTCTTTG-3′;IL-1β: forward 5′-TCCAGGATGAGGACATGAGCAC-3′ and reverse 5′-GAACGTCACCCAGCAGGTTA-3′;IL-6: forward 5′-CCACTTCACAAGTCGGAGGCTTA-3′ and reverse 5′-CCAGTTTGGTAGCATCCATCATTTC-3′. GAPDH: forward 5′ - GGCATTGCTCTCAATGACAA - 3′ and Reverse 5′ - GGTGGTCCAGGGTTTCTTAC - 3′.

### 2.4 Western blot analysis

Murine hepatic tissues were lysed using a commercial lysis buffer, and total protein concentration was determined with a BCA Protein Assay Kit (Beyotime, China). Equal amounts of protein samples were separated by sodium dodecyl sulfate-polyacrylamide gel electrophoresis (SDS-PAGE) on a Bio-Rad Mini-PROTEAN system. The resolved proteins were then electrotransferred onto polyvinylidene fluoride (PVDF) membranes (Bio-Rad). Membranes were blocked with 5% non-fat milk in Tris-buffered saline with Tween 20 (TBST) for 1 hour at 37°C, followed by overnight incubation with primary antibodies at 4°C. The primary antibodies used were: anti-NLRP3 (Immunoway, YT5382, 1:1000), anti-ASC(Cell Signaling Technology, 67824, 1:1000), anti-IL-18(Wanlei, WL01127, 1:1000), anti-Cleaved-GSDMD(Wanlei, WL01127, 1:1000), anti-Cleaved-Caspased-1 (abcam, ab179515, 1:1000), and anti-Cleaved-IL-1β (abcam, ab179515, 1:1000), and anti-GAPDH (Proteintech, 60004-1-Ig, 1:5000) as a loading control. After washing, membranes were incubated with horseradish peroxidase (HRP)-conjugated secondary antibodies (Beyotime goat anti-rabbit IgG and Proteintech goat anti-mouse IgG, both diluted 1:2000) for 1 hour at room temperature. Protein bands were visualized using an enhanced chemiluminescence (ECL) detection system and quantified with ImageJ software (Version 1.50i). Relative protein expression levels were calculated as the ratio of target protein intensity to GAPDH intensity.

### 2.5 Immunohistochemical staining

Hepatic tissue sections were subjected to dewaxing and rehydration procedures, after which antigen retrieval was performed using citrate buffer in a pressure cooker. Once cooled to room temperature, the sections were incubated with 3% hydrogen peroxide for 15 minutes and subsequently rinsed three times with PBST. The tissue sections were then blocked with 5% BSA at room temperature for 20 minutes, followed by overnight incubation at 4°C with the primary antibody—anti-F4/80 (1:500, WLH2545, Wanlei). After three additional washes with PBST, the sections were incubated with HRP-conjugated secondary antibodies (HRP-labeled Goat Anti-Rabbit IgG (H+L), Beyotime, 1:700) at room temperature for 1 hour. Following further washing, the sections were treated with DAB (Maxim) for color development and counterstained with hematoxylin. After dehydration, the sections were mounted with neutral balsam, and images were acquired using a pathological slide scanner.

For the quantification of F4/80-positive macrophages, five non-overlapping fields per section were randomly selected and captured at ×100 magnification using a pathological slide scanner. F4/80+ cells exhibiting clear brown DAB staining and typical macrophage morphology were manually counted in each field by two independent investigators who were blinded to the group assignments. The average count from the two observers for the five fields was calculated and used as the final value for each sample, thereby minimizing subjective bias in the quantification process.

### 2.6 TUNEL staining assay

Hepatic tissue apoptosis was evaluated using the TUNEL Apoptosis Assay Kit (cat. no. C1088; Beyotime Institute of Biotechnology), following the protocol outlined below: Paraffin-embedded tissue sections were first deparaffinized in xylene and rehydrated through a gradient of ethanol solutions at room temperature. After rinsing with phosphate-buffered saline (PBS), the sections were fixed in 1% paraformaldehyde for 15 minutes at ambient temperature, then treated with proteinase K working solution at 37°C for 10 minutes. Next, the sections were incubated with TUNEL reaction mixture in the dark at 37°C for 60 minutes. Nuclear counterstaining was performed with 4’,6-diamidino-2-phenylindole (DAPI) for 5 minutes at room temperature, followed by mounting with anti-fade medium (Beijing Solarbio Science & Technology Co., Ltd.). Finally, five randomly selected fields (×100 magnification) were photographed using an inverted fluorescence microscope (Olympus IX71; Olympus Corporation).

### 2.7 Biochemical indicators of hepatic function

Alanine aminotransferase (ALT; C009-2-1) and aspartate aminotransferase (AST; C010-2-1) levels were measured using commercial assay kits from Nanjing Jiancheng Bioengineering Institute, following the manufacturer’s protocols. The assays were performed with an Olympus AU400 automatic biochemical analyzer (Olympus, Tokyo, Japan).

### 2.8 RNA sequencing and data analysis

Total RNA was extracted from liver tissues of the sepsis group (n = 3) and the sepsis + TCDCA group (n = 3). The RNA samples were submitted to Novogene Co., Ltd. (Beijing, China) for cDNA library preparation and sequencing using the Illumina NovaSeq 6000 platform. Raw sequencing reads were aligned to the Homo sapiens reference genome using HISAT2 (v2.0.5), and gene-level read counts were obtained with FeatureCounts (v1.5.0-p3). Gene expression levels were quantified as fragments per kilobase of transcript per million mapped reads (FPKM).Differential expression analysis was performed using the DESeq2 package (v1.20.0) in R. Genes with an FDR-adjusted *p*-value (padj) < 0.05 were identified as differentially expressed genes (DEGs). Kyoto Encyclopedia of Genes and Genomes (KEGG) pathway analyses, were subsequently carried out on the DEGs.

### 2.9 Statistical analysis

Statistical analysis of the experimental data was performed using SPSS 13.0 software. Measurement data were presented as mean ± standard deviation (SD). <mark>Prior to parametric testing, the normality of data distribution was confirmed using the Shapiro-Wilk test, and the homogeneity of variances was verified using Levene’s test. For comparisons between two groups, the independent samples t-test was utilized, whereas one-way analysis of variance (ANOVA) (following confirmation of variance homogeneity) was employed for comparisons among multiple groups. A value of *P* < 0.05 was considered statistically significant.

## 3 Results

### 3.1 TCDCA protected against SALI

**Figure 1:**
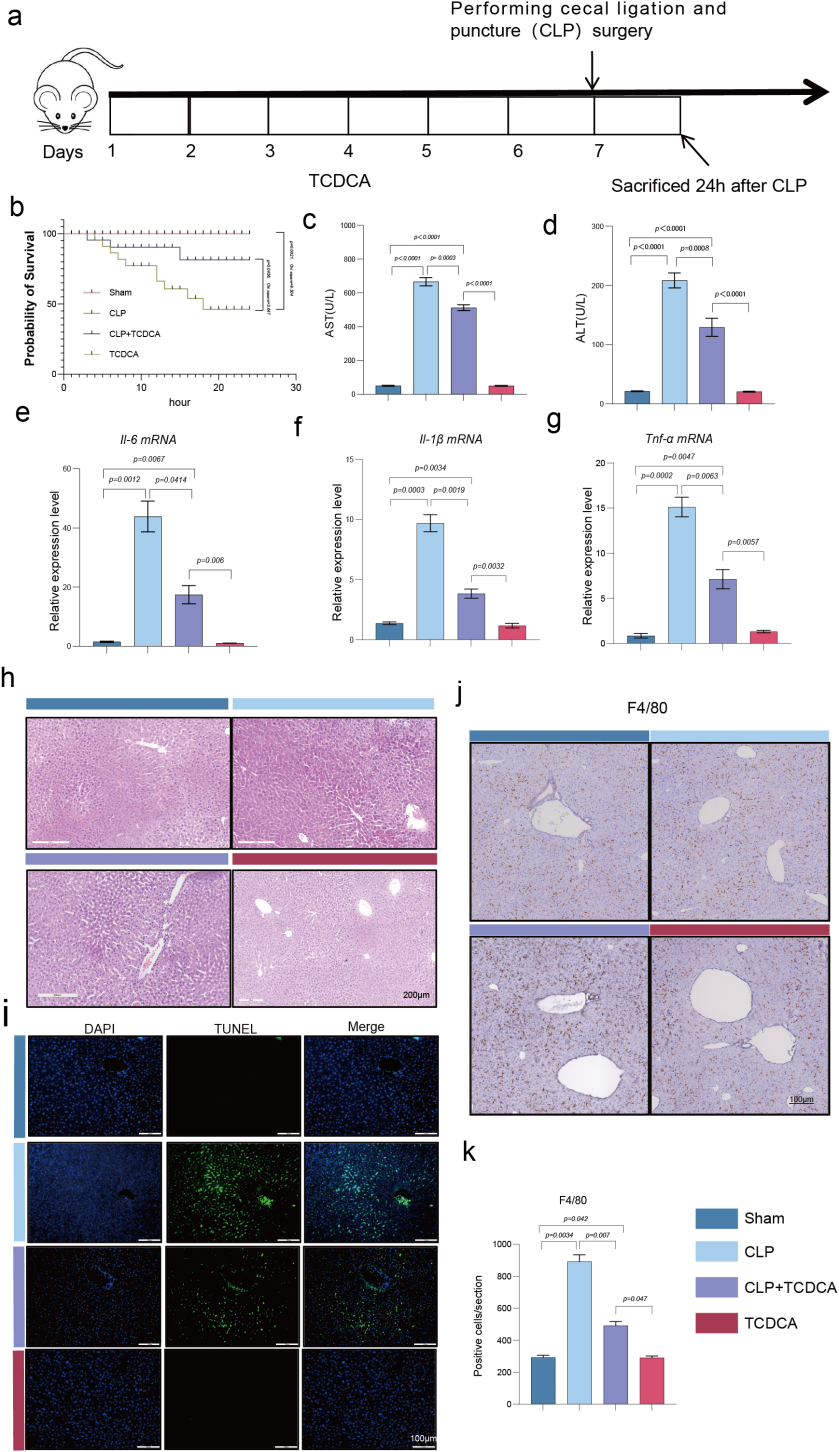
TCDCA alleviated hepatic injury and inflammation in CLP-induced SALI. **(a)** Schematic diagram of the experimental design. **(b)** Kaplan-Meier survival curves of mice over 24 hours following CLP surgery (Sham, n=15; CLP, n=15; CLP+TCDCA, n=15). Statistical significance was determined by the log-rank test. **(c, d)** Serum levels of aspartate aminotransferase (AST, c) and alanine aminotransferase (ALT, d), markers of hepatic injury. **(e–g)** Hepatic mRNA expression levels of the pro-inflammatory cytokines Il6 **(e)**, Il1b **(f)**, and Tnf **(g)**, analyzed by qPCR. **(h)** Representative images of H&E-stained liver sections, showing histopathological changes. **(i)** Representative images of TUNEL staining (green) for detecting apoptotic hepatocytes; nuclei are counterstained with DAPI (blue). **(j, k)** Representative images of immunohistochemical staining for F4/80-positive macrophages and the corresponding quantitative analysis. Scale bars in **(h–j)** represent 100 μm. Data in panels c-g are presented as mean ± SEM (n = 6 per group, randomly selected from survivors for statistical comparability). Statistical significance was determined by one-way ANOVA with appropriate *post-hoc* tests. Images were captured using an Olympus CX30 light microscope.

To investigate the protective effect of TCDCA against sepsis-associated acute liver injury, we established a mouse model of sepsis using cecal ligation and puncture (CLP), as illustrated in **Figure 1a**. Survival analysis performed using the Kaplan-Meier method revealed that the CLP group exhibited a significantly lower survival probability within 24 hours compared to the Sham group (**Figure 1b**, p < 0.01, log-rank test). TCDCA administration, however, markedly improved the survival rate of septic mice (*p* < 0.01 vs. CLP group, log-rank test) (**Figure 1b**). TCDCA administration, however, markedly improved the survival rate of septic mice. Assessment of liver function indicators showed that AST (**Figure 1c**) and ALT (**Figure 1d**) levels were significantly elevated in the CLP group relative to the Sham group, while TCDCA treatment substantially reduced the concentrations of these enzymes. Quantitative PCR analysis further demonstrated that the mRNA expression levels of the pro-inflammatory cytokines IL-6 (**Figure 1e**), TNF-α (**Figure 1g**), and IL-1β (**Figure 1f**) were significantly increased in the CLP group, and these increases were effectively attenuated by TCDCA intervention. Histopathological examination via H&E staining revealed severe hepatic damage in the CLP group, characterized by disruption of the lobular architecture, edema, inflammatory cell infiltration, and hemorrhage—all of which were notably ameliorated by TCDCA treatment (**Figure 1h**). Consistent with these findings, TUNEL staining indicated a markedly higher rate of hepatocyte apoptosis in the CLP group compared to the Sham and TCDCA groups, with TCDCA significantly reducing apoptosis (**Figure 1i**). In addition, immunohistochemical analysis of F4/80+ macrophages showed that sepsis induction led to a substantial increase in macrophage infiltration in the liver, an effect that was reversed by TCDCA administration (**Figures 1j, k**).

### 3.2 TCDCA protected against SALI via TGR5

**Figure 2:**
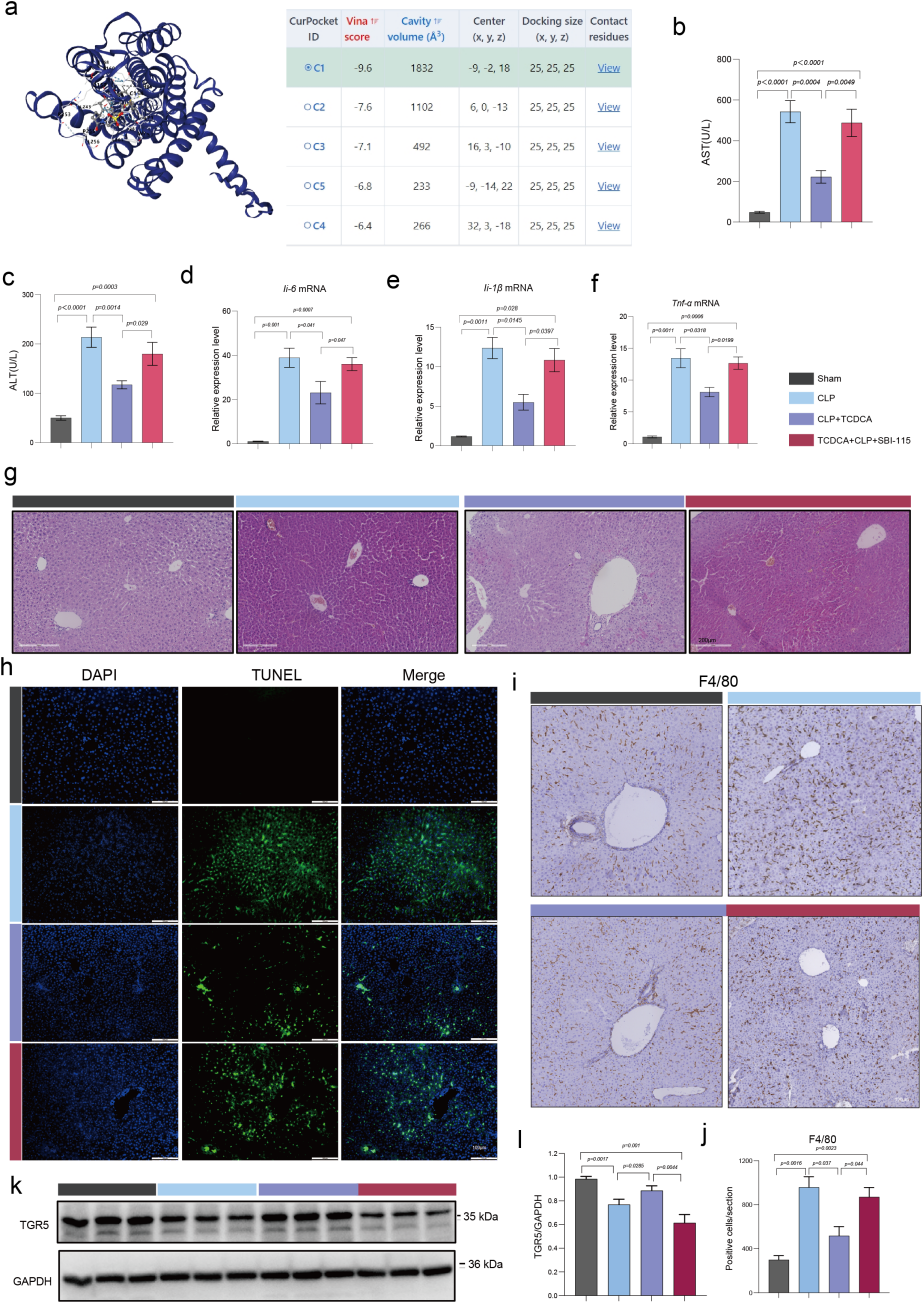
TCDCA-mediated protection against SALI requires TGR5 activation. **(a)** Molecular docking model illustrating the interaction between TCDCA and the TGR5 receptor, with a calculated binding affinity of -9.6 kcal/mol. **(b, c)** Serum levels of ALT **(b)** and AST **(c)**. **(d–f)** Hepatic mRNA expression levels of Il6 **(d)**, Tnf **(e)**, and Il1b **(f)**, measured by qPCR. **(g)** Representative photomicrographs of H&E-stained liver sections. **(h)** Representative images of TUNEL staining (green) for apoptotic hepatocytes; nuclei are counterstained with DAPI (blue). **(i, j)** Representative images of immunohistochemical staining for F4/80-positive macrophages and the corresponding quantitative analysis. **(k)** Representative Western blot bands showing TGR5 protein expression, with GAPDH serving as the loading control. **(l)** Densitometric quantification of TGR5 protein levels normalized to GAPDH. Scale bars in **(g–i)** represent 100 μm. Data in panels b-f and k are presented as mean ± SEM (n = 6 per group). Images were captured using an Olympus CX30 light microscope.

TGR5, also known as G - protein coupled receptor 131 (GPR131), is an integral member of the GPCR superfamily, initially identified as a bile acid receptor (15). Leveraging AutoDockTools software for molecular docking analysis, the reaction free energy for the chlorination of TCDCA was determined to be -9.6 kcal/mol, as depicted in **Figure 2a**. To explore the functional role of TGR5, we conducted a mouse study. In the TCDCA-treated group, AST and ALT levels were reduced; notably, administration of SBI-115 led to a significant increase in these enzyme levels (**Figures 2b, c**). qPCR was used to evaluate the expression of hepatic inflammatory cytokines. Compared with the CLP group, the CLP+TCDCA group showed significantly lower expression of IL-6, TNF-α, and IL-1β, whereas the addition of SBI-115 to TCDCA treatment resulted in a marked elevation of these inflammatory factors (**Figures 2d–f**). H&E staining revealed that, in contrast to TCDCA-treated samples, SBI-115 administration caused disruption of hepatic lobular architecture, accompanied by edema, inflammatory cell infiltration, and hepatic hemorrhage (**Figure 2g**). TUNEL staining of liver sections showed minimal hepatocyte apoptosis in the CLP+TCDCA group compared with the CLP group, but this protective effect was reversed by SBI-115, which exacerbated apoptosis (**Figure 2h**). Analysis of hepatic macrophage responses demonstrated that TCDCA treatment reduced the number of F4/80+ macrophages in the CLP+TCDCA group, while SBI-115 reversed this effect, leading to increased macrophage counts (**Figures 2i, j**). Western blot analysis was performed to assess TGR5 protein expression: compared with the sham group, CLP significantly downregulated TGR5 expression; TCDCA upregulated TGR5 expression relative to the CLP group, and this effect was reversed by SBI-115 (**Figures 2k, l**).

### 3.3 The mechanism of the function of TCDCA

**Figure 3:**
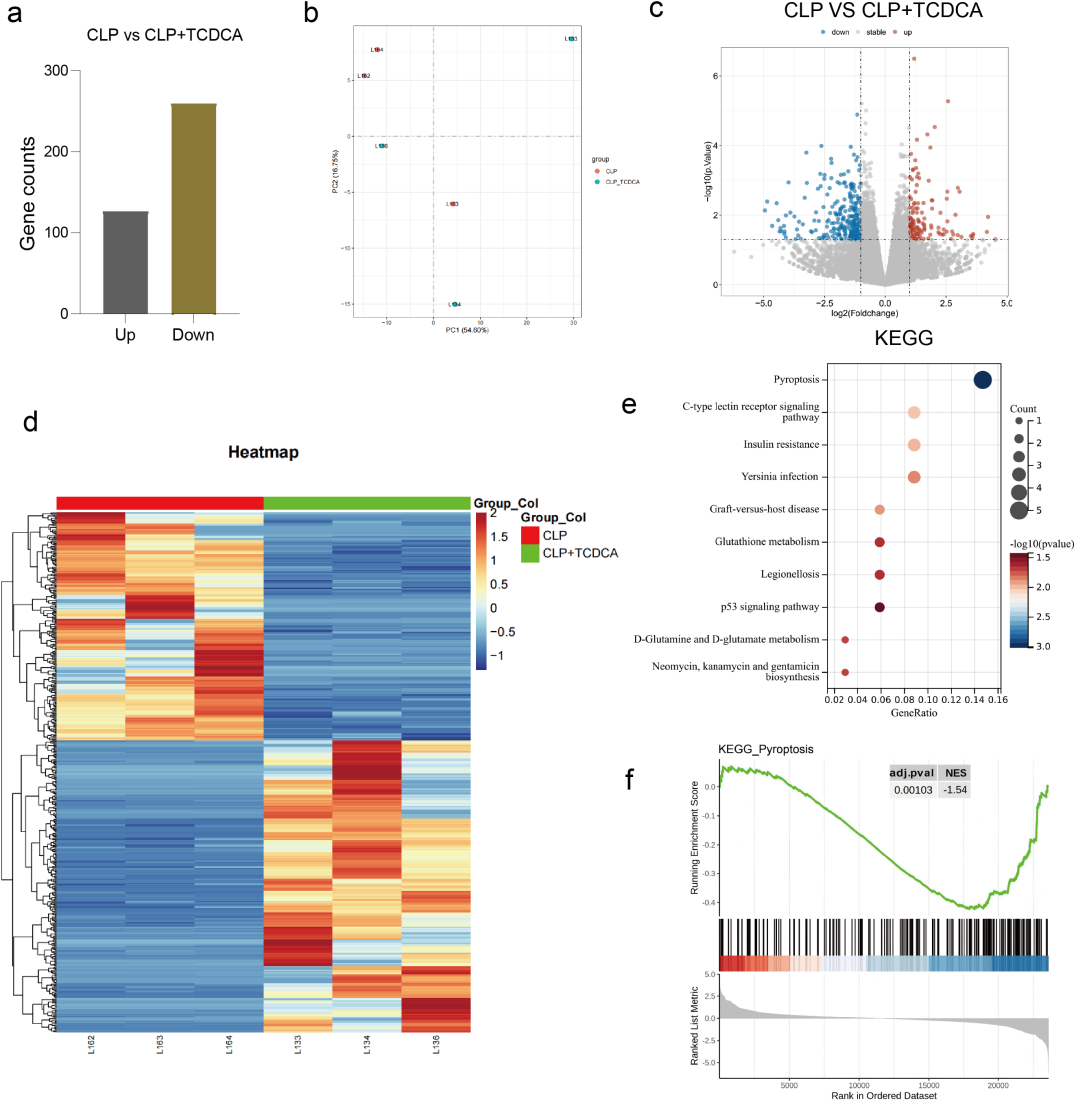
Transcriptomic analysis identified pyroptosis as a key pathway regulated by TCDCA. **(a)** Principal component analysis (PCA) plot of global gene expression profiles, demonstrating clear separation between the CLP (n=3) and CLP+TCDCA (n=3) groups. **(b)** Bar graph summarizing the total number of differentially expressed genes (DEGs) identified in the CLP+TCDCA group compared to the CLP group (350 total; 223 upregulated, 127 downregulated; *P* < 0.05). **(c)** Volcano plot visualizing the DEGs. Significantly upregulated genes are shown in red, downregulated genes in blue, and non-significant genes in grey. **(d)** Heatmap generated by hierarchical clustering analysis, displaying the expression patterns of the identified DEGs across individual samples. Red indicates high expression and blue indicates low expression. **(e)** Bar plot of Kyoto Encyclopedia of Genes and Genomes (KEGG) pathway enrichment analysis for the DEGs. The most significantly enriched pathways are shown, with the pathway for pyroptosis highlighted. **(f)** Gene Set Enrichment Analysis (GSEA) confirmed that the pyroptosis pathway was significantly enriched in the CLP group compared to the CLP+TCDCA group.

Transcriptome analysis was performed to investigate the mechanism underlying TCDCA’s function. Principal component analysis (PCA) was applied to assess the global gene expression profiles of the CLP group (n=3) and CLP+TCDCA group (n=3), showing a clear separation between the two groups along the first principal component (PC1) (**Figure 3a**). According to *p*-value (padj) < 0.05, statistical analysis of differentially expressed genes revealed that TCDCA treatment induced significant changes in 350 genes, with 223 upregulated and 127 downregulated (**Figure 3b**). Volcano plots were used to visualize significantly differentially expressed genes (**Figure 3c**). Hierarchical clustering analysis identified differentially expressed genes, which were displayed in a heatmap with red indicating high expression and blue indicating low expression (**Figure 3d**). KEGG pathway enrichment analysis showed that these differentially expressed genes were primarily enriched in pathways such as lignin biosynthesis and pyroptosis (**Figure 3e**). In addition to the overrepresentation analysis, Gene Set Enrichment Analysis (GSEA) confirmed that the pyroptosis pathway was significantly enriched in the CLP group compared to the CLP+TCDCA group, further validating the involvement of pyroptosis in the protective mechanism of TCDCA (**Figure 3f**).

### 3.4 TCDCA inhibited pyroptosis to protect against SALI via TGR5

**Figure 4:**
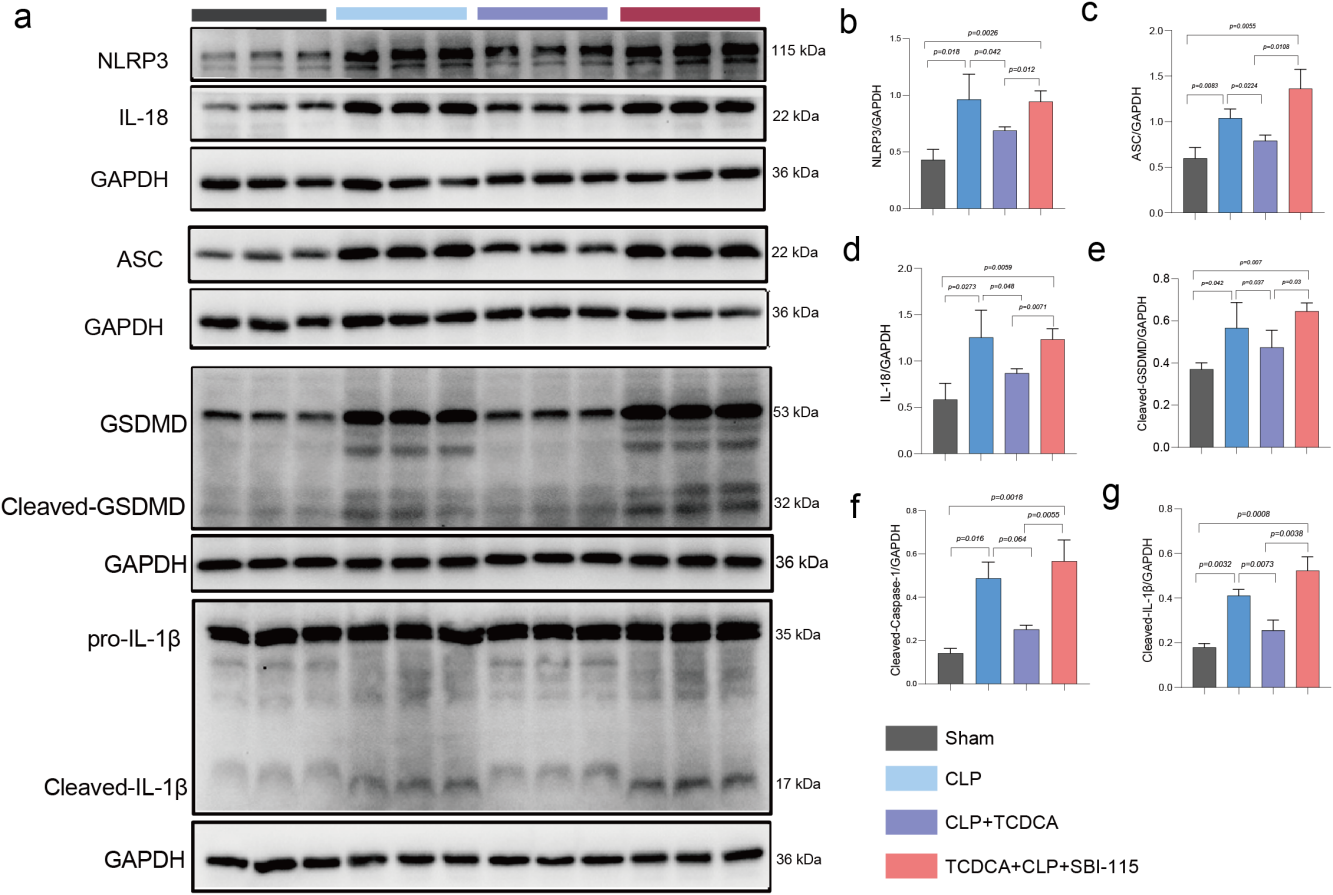
TCDCA inhibited pyroptosis via TGR5. **(a)** Representative Western blot images of key pyroptosis-related proteins in hepatic tissues: NLRP3, ASC, Cleaved Caspase-1 (C-Casp1), Cleaved GSDMD (C-GSDMD), mature IL-18, and Cleaved IL-1b (C-IL-1b). GAPDH was used as a loading control. **(b–g)** Densitometric quantification of protein expression levels for **(b)** NLRP3, **(c)** ASC, **(d)** C-Casp1, **(e)** C-GSDMD, **(f)** IL-18, and **(g)** C-IL-1b. Data are normalized to GAPDH and presented as mean ± SEM (n = 3 per group). Statistical. Bar graphs show quantitative data with statistical significance indicated by p-values.

Western blot analysis was performed to verify whether the protective effect of TCDCA on sepsis-associated acute liver injury (SALI) relies on TGR5-mediated suppression of pyroptosis. In the hepatic tissues of CLP-induced mice, the expression levels of pyroptosis-related proteins—including NLRP3, ASC, IL-18, cleaved GSDMD, cleaved caspase-1, and cleaved IL-1β—were significantly elevated. TCDCA treatment, however, downregulated the expression of these proteins, while subsequent administration of SBI-115 reversed this inhibitory effect, leading to increased expression of the aforementioned pyroptosis markers (**Figures 4a-g**). Collectively, these results indicate that TCDCA exerts a protective effect against SALI by inhibiting pyroptosis activation through TGR5.

## 4 Discussion

SALI represents a life-threatening complication in critically ill patients, characterized by a rapid decline in hepatic function and an associated high mortality rate (16). Despite advancements in supportive care and antimicrobic therapy, SALI remains a significant clinical challenge, underscoring the urgent need for novel therapeutic strategies (17). This study elucidated the protective role of TCDCA in SALI and uncovers its underlying molecular mechanisms, providing valuable insights into the pathophysiology of sepsis and potential therapeutic targets.

The protective effects of TCDCA against SALI were comprehensively evaluated using a battery of biochemical, histological, and molecular assays. Serum levels of AST and ALT were markedly elevated in the sepsis group, reflecting severe hepatic injury. Notably, TCDCA treatment led to a significant reduction in these enzyme levels, indicating improved hepatocellular integrity. This finding was further supported by the decreased expression of pro-inflammatory cytokines, including IL-6, TNF-α, and IL-1β. Histopathological examination revealed that TCDCA treatment attenuated Hepatic lobular disorganization, inflammatory cell infiltration, and hemorrhage, restoring the normal architecture of the hepatic. These results were consistent with previous studies demonstrating the anti-inflammatory and cytoprotective properties of bile acid metabolites in sepsis, reinforcing the potential of TCDCA as a novel therapeutic agent for SALI (18).

A key discovery of this study is the essential role of the TGR5 in mediating the protective effects of TCDCA against SALI. Functional studies using the specific TGR5 antagonist SBI-115 demonstrated that blocking TGR5 abolished the protective effects of TCDCA, as evidenced by the restoration of elevated AST and ALT levels, increased expression of pro-inflammatory cytokines, and exacerbation of hepatic histopathological changes. These findings provide compelling evidence that TGR5 is the primary target through which TCDCA exerts its hepatoprotective effects. Although the present study confirms that TCDCA inhibits hepatocyte pyroptosis via TGR5, the immediate downstream signaling events warrant further investigation. Based on established literature of TGR5 signaling, TGR5 has been found that ischemic preconditioning can activate the TGR5 signaling pathways, such as the TGR5/cAMP/PKA, TGR5/p38 MAPK and TGR5/NF-κB signaling pathways (19). This activation can reduce the generation of damage factors, including oxidative stress, inflammatory response and apoptosis, enhance the anti-stress ability of cells, inhibit the occurrence of apoptosis, and thus alleviate liver ischemia-reperfusion injury and the apoptotic response (20). Elucidating the precise contribution of these downstream effectors will be a central focus of our subsequent research. This results suggest that activation of TGR5 by TCDCA initiates a signaling cascade that enhances macrophage anti-inflammatory activity and restores immune homeostasis, thereby mitigating sepsis-induced Hepatic injury (21, 22).

Our findings highlight TCDCA’s protective role via TGR5, which invites comparison with other well-studied bile acid metabolites such as lithocholic acid (LCA) and deoxycholic acid (DCA). While LCA and DCA are potent TGR5 agonists, they also serve as high-affinity ligands for the farnesoid X receptor (FXR) (23). The anti-inflammatory effects of LCA and DCA are often attributed to FXR activation, which can suppress NF-κB signaling and subsequent NLRP3 inflammasome priming (24). In contrast, our data suggest that TCDCA’s primary mechanism in alleviating SALI is through TGR5 activation, leading to direct inhibition of the NLRP3 inflammasome and pyroptosis execution, independent of FXR (25). This TGR5-centric pathway may offer a more rapid, membrane receptor-mediated cytoprotective signal, distinct from the genomic actions of FXR (26). This distinction potentially positions TCDCA as a more specific therapeutic agent for acute inflammatory conditions like SALI, where swift intervention is critical.

Transcriptomic analysis was employed to gain a comprehensive understanding of the molecular changes induced by TCDCA treatment. Pathway analysis revealed significant enrichment of genes involved in lignin biosynthesis and pyroptosis, suggesting potential mechanisms underlying the protective effects of TCDCA. Pyroptosis, a form of programmed inflammatory cell death, has emerged as a critical factor in the pathogenesis of sepsis-induced organ injury (27, 28). By inhibiting pyroptosis, TCDCA reduced the release of pro-inflammatory cytokines and damage-associated molecular patterns (DAMPs), thereby interrupting the vicious cycle of inflammation and tissue damage (29). These findings provide novel insights into the molecular mechanisms by which TCDCA protected against SALI and highlight the potential of targeting pyroptosis as a therapeutic strategy for sepsis.

Despite these significant findings, several limitations of the study should be acknowledged. Such as, the current study was conducted using a murine model of sepsis, and the translation of these findings to human patients requires further investigation. Clinical trials are essential to validate the efficacy and safety of TCDCA in human sepsis patients and to determine the optimal dosing regimen and treatment duration. It is also important to note that while we used a pharmacological antagonist (SBI-115) to demonstrate TGR5 dependency, future studies employing TGR5 knockout mouse models would provide definitive genetic evidence to confirm the specificity of TCDCA’s action.

While this study identified TGR5 as the key receptor mediating the protective effects of TCDCA, the downstream signaling pathways remain incompletely characterized. The precise molecular events linking TGR5 activation to the inhibition of pyroptosis mechanisms were not fully understood. Future studies should employ advanced proteomic and genetic techniques, such as gene knockout and overexpression models, to elucidate the detailed signaling pathways involved.

Although the present study confirms that TCDCA inhibits hepatocyte pyroptosis via TGR5, the downstream signaling events warrant further investigation. Previous studies have shown that TGR5 activation could trigger multiple intracellular pathways, including the cAMP/PKA, p38 MAPK, and NF-κB cascades, which were known to modulate inflammasome activation and pyroptosis. For instance, cAMP-elevating agents have been reported to suppress NLRP3 inflammasome assembly via PKA-mediated phosphorylation of NLRP3 (30). Similarly, TGR5-mediated inhibition of NF-κB could reduce the transcription of pyroptosis-related components such as pro-IL-1β and NLRP3 (10). Thus, it is plausible that TCDCA activates one or more of these TGR5-dependent pathways to exert its anti-pyroptotic effects. Future studies using pathway-specific inhibitors or genetic models will help delineate the precise signaling mechanisms underlying TCDCA’s protection against SALI.

In conclusion, this study demonstrated that TCDCA protected against SALI by suppressing hepatocyte pyroptosis via TGR5. These findings provided novel insights into the pathophysiology of sepsis and identify TCDCA as a promising therapeutic candidate for SALI. However, further research is needed to address the limitations of the current study and to translate these findings into clinical practice. Future studies should focus on validating the efficacy and safety of TCDCA in human sepsis patients, elucidating the detailed molecular mechanisms of action, and exploring the potential of combination therapies to improve the outcomes of sepsis patients with Hepatic injury.

## Data Availability

The datasets presented in this study can be found in online repositories. https://www.ncbi.nlm.nih.gov/, PRJNA1265619.
